# Rapid Phylogeographic Inference of Regional Epidemic Dynamics for Routine Genomic Surveillance

**DOI:** 10.21203/rs.3.rs-10631496/v1

**Published:** 2026-08-11

**Authors:** Leke Lyu, Nicola F. Müller, Zewen Liu, Lambodhar Damodaran, Wei Jin, Max S. Y. Lau

**Affiliations:** a. Department of Biostatistics and Bioinformatics, Emory University, Atlanta, GA 30322;; b. Division of HIV, Infectious Diseases & Global Medicine, Department of Medicine, University of California San Francisco, San Francisco, CA 94143;; c. Department of Computer Science, Emory University, Atlanta, GA 30322;; d. Department of Biology, Emory University, Atlanta, GA 30322;

**Keywords:** Viral evolution, Genomic epidemiology, Phylogeographic, Graph neural network, Pandemic control

## Abstract

Genomic surveillance has become a cornerstone of epidemic response, yet translating the large phylogenies generated by modern sequencing efforts into actionable regional epidemic intelligence remains beyond the reach of most public health systems. Existing phylogeographic methods are too computationally demanding for routine deployment, creating a bottleneck between genomic data generation and epidemiological insight. Here, we introduce STEPHY, a readily deployable graph neural inference toolkit that enables rapid estimation of regional epidemic dynamics directly from large viral phylogenies.

By representing phylogenies as region-level graphs and integrating information across connected regional outbreaks, STEPHY jointly estimates region-specific reproduction numbers, recovery rates, source-sink roles, and the most likely index location — epidemiological quantities that directly inform geographically targeted surveillance and intervention. STEPHY is adaptable to diverse pathogens and surveillance settings through locally tailored simulations.

We validate STEPHY through simulation studies across complex metapopulation systems, demonstrating accurate recovery of the regional epidemiological quantities and showing that jointly modeling cross-regional dependencies substantially outperforms independent region-by-region analysis. We apply STEPHY to five densely sampled SARS-CoV-2 lineage phylogenies from Denmark, one of the most comprehensively sequenced national epidemics on record, spanning the Alpha, Delta, and Omicron waves under markedly different epidemic conditions. STEPHY consistently identifies Hovedstaden as the dominant source of viral dissemination, with its source role becoming increasingly pronounced from Alpha through Omicron, revealing a progressive strengthening of spatial transmission heterogeneity across successive variant waves. Inference across phylogenies containing 571 to 7,177 sequences requires only milliseconds, confirming that STEPHY scales to the demands of real-world genomic surveillance.

Together, these results establish STEPHY as a scalable and readily deployable toolkit for regional epidemic inference and response using routine genomic surveillance data.

## Introduction

Pathogen genomes encode a record of evolutionary and transmission processes in host populations (1–4). The COVID-19 pandemic marked a turning point for genomic epidemiology, showing that large-scale, real-time sequencing can become central to outbreak response by enabling rapid tracking of key mutations (5, 6), variant emergence (7, 8), and lineage replacement (9). When integrated with epidemiological and individual-level metadata, genomic data can further characterize transmission patterns (10), including differences in transmissibility (11), prevalence (12), and dissemination routes (13–16), and identify demographic factors shaping these patterns (17). These insights inform our understanding of epidemic spread and the design of effective control strategies (18). This momentum has extended to later outbreaks, including mpox (19–21) and highly pathogenic avian influenza (22), underscoring the growing importance of genomic epidemiology for emerging infectious diseases (23). However, the increasing volume and spatial detail of genomic surveillance data have outpaced the scalability of many existing phylodynamic models, creating a bottleneck between genomic data generation and actionable regional epidemic intelligence (24, 25).

A major goal of genomic epidemiology is phylogeographic inference — characterizing how pathogens spread across interconnected populations (e.g., identifying transmission hotspots and regions driving spatial spread) to guide geographically targeted surveillance and control strategies (26). Structured models within Bayesian phylodynamic frameworks have become a gold-standard approach for phylogeographic inference (27, 28). By assigning pathogen lineages to discrete geographic populations, these models can reconstruct pathogen movement across regions and estimate region-specific transmission dynamics. Two major frameworks (29) are commonly used: structured coalescent models, which trace lineage ancestry backward in time through migration and coalescence (30), and multi-type birth-death models, which model epidemic dynamics forward in time through transmission, removal, sampling, and movement between populations (31–33). Exact structured coalescent approaches explicitly model ancestral migration histories, enabling mechanistic phylogeographic inference but becoming computationally expensive as the number of discrete regions increases (34). Later approximations (35–37), including marginal structured coalescent methods such as MASCOT (38), improved scalability by avoiding explicit reconstruction of every migration event. In parallel, multi-type birth-death models have been refined to improve numerical stability and scalability for larger empirical datasets (39), with newer approaches incorporating stochastic mapping and particle filtering to infer ancestral state histories and latent population trajectories (40). Despite these advances, both frameworks are still computationally challenging for routine, high-resolution phylogeographic analyses (41).

Simulation-based inference approaches, such as approximate Bayesian computation (ABC), have been developed as alternatives to likelihood-based phylodynamic methods (42). ABC estimates parameters by comparing summary statistics from observed data with those generated under simulated epidemiological scenarios, without requiring explicit likelihood evaluation (43). More recently, machine-learning approaches have used large collections of simulated phylogenies to train neural networks that learn a direct mapping from phylogenetic data to epidemiological parameters. Once trained, the learned mapping can then be reused to perform rapid inference on new phylogenies through a single forward pass. Early studies showed that deep neural networks can accurately perform model selection and parameter inference directly from phylogenetic trees while substantially reducing computational cost relative to conventional Bayesian approaches (44, 45). Later work improved phylogenetic tree representations and reduced training-data requirements (46). Exploratory studies showed the feasibility of applying deep learning to phylogeographic inference (47). More recent developments have introduced flexible software frameworks for constructing customized machine-learning pipelines for phylogenetic analyses (48) and extended simulation-based learning toward inference of time-varying epidemic dynamics (49). Yet, while these advances establish machine learning as a promising foundation for scalable phylodynamic inference, their application to geographically structured populations remains limited. In particular, existing simulation-based deep-learning frameworks do not explicitly learn, directly from phylogenies, how phylogenetic signals across regions jointly encode spatial epidemic dynamics.

In geographically structured populations, epidemic dynamics emerge from interactions among multiple regions, where local evolutionary histories are shaped by both within-region transmission and movement between connected populations. As the number of regions increases, these interdependencies become increasingly complex. Graph-based representations provide a natural framework for modeling these systems by representing regions as nodes and relationships between regions as edges (50). Building on this representation, graph neural networks (GNNs) are well suited for structured epidemic systems because message passing allows each region to incorporate relational information from connected regions (51). In recent years, the increasing adoption of GNNs across epidemiological applications, including outbreak detection (52), disease surveillance (53, 54), epidemic forecasting (55–57), and intervention planning (58–61), has demonstrated their potential for learning complex transmission patterns in infectious disease systems.

Here, we present STEPHY (**S**patial **T**ransmission **E**stimation from **PHY**logenies), a graph neural inference framework for the rapid estimation of regional epidemic dynamics directly from large phylogenies. Taking time-calibrated phylogenies as input, STEPHY introduces a new graph representation tailored to geographically structured phylogenies. Specifically, STEPHY encodes each phylogeny as a region-level graph in which nodes capture region-specific subtree structure and edges encode temporal synchronization between regional outbreaks — a representation designed to retain the coupling signals among regional epidemics encoded in the phylogeny. Node features summarize each region’s portion of the phylogeny using the Compact Bijective Ladderized Vector (CBLV), which retains subtree topology and branch lengths in a format suitable for neural-network analysis (44, 47), together with additional phylogenetic summary statistics (47). Edge features are derived by adapting dynamic time warping (DTW), a method previously used to compare asynchronous epidemic time series (63, 64), into a directed edge-feature encoder for phylogenies, capturing temporal lead-lag relationships between regional sampling-time profiles that reflect cross-regional seeding histories. Through edge-aware message passing, STEPHY integrates information across regions, allowing each region to incorporate signals from temporally related outbreaks while preserving its own phylogenetic characteristics. Together, these components enable the joint estimation of region-specific reproduction numbers, recovery rates, and source-sink scores that summarize each region’s role as a source or sink of transmission (65, 66), as well as the identification of the most likely index location. In simulation benchmarks, STEPHY substantially improves prediction accuracy and produces narrower prediction intervals relative to an otherwise matched baseline that treats each region independently, demonstrating that phylogenies carry cross-regional coupling signals beyond what independent subtree analysis captures.

To evaluate STEPHY on empirical data, we apply it to SARS-CoV-2 genomic surveillance data from Denmark, which achieved some of the highest sequencing coverage worldwide during the COVID-19 pandemic (18, 67). This extensive dataset has previously supported large-scale phylodynamic analyses of clade-level reproduction numbers (18, 44), providing a well-characterized empirical setting in which to evaluate how STEPHY extends inference to geographically structured epidemic dynamics. We train a Denmark-specific model to characterize transmission across the country’s five administrative regions and apply it to five major viral lineages spanning the Alpha, Delta, and Omicron waves. These lineages encompass markedly different epidemiological settings, including changes in non-pharmaceutical interventions, population immunity, vaccination coverage, and transmission intensity (68, 69). STEPHY jointly estimates region-specific epidemiological quantities, including reproduction numbers and source-sink roles, while requiring only milliseconds to analyze large phylogenies, including one lineage with more than 7,000 tips. Across all five lineages, STEPHY consistently identifies Hovedstaden, the capital region, as the strongest source of viral dissemination to other regions. This source role becomes increasingly pronounced from Alpha through Delta to Omicron. To assess robustness to phylogenetic uncertainty, we repeat inference across 40 bootstrap replicate trees for each lineage and find that the resulting estimates are broadly consistent with those obtained from the maximum-likelihood trees. STEPHY can be adapted to diverse pathogens and surveillance settings by tailoring simulations to local population structure and epidemiological scenarios.

Together, STEPHY provides a scalable, adaptable, and readily deployable framework for regional epidemic inference and response using routine genomic surveillance data.

## Results

### Outbreak Simulation and Epidemiological Inference Targets

STEPHY (Spatial Transmission Estimation from PHYlogenies) is a graph neural inference framework that learns a direct mapping from time-calibrated phylogenies to region-specific epidemiological quantities (Fig. 1). We trained STEPHY using simulated outbreaks in which the target epidemiological quantities were known by construction, enabling amortized inference on new phylogenies through a single forward pass. To generate these training data, we simulated stochastic outbreaks across n discrete regions using an SIR metapopulation model, with parameters sampled to span a broad range of spatial epidemic regimes. We chose the SIR model as a minimal yet general epidemic model because its stochastic events capture the core processes required for this study: infection within regions, migration between regions, recovery, and sampling into the observed phylogeny. For each region $i,S_{i}(t),I_{i}(t),R_{i}(t)$, and ${\text{Smp}}_{i}(t)$ denote the numbers of susceptible, infectious, recovered, and sampled individuals at time t, respectively. Outbreak trajectories are generated using the Gillespie stochastic simulation algorithm (70), which sequentially samples these events according to their rates. During the simulation, infection events define the transmission history, while sampling events determine which infections are observed (62). The resulting transmission history is then converted into a time-calibrated phylogeny, with sampled infections forming the tips (Fig. 1a).

Each simulated phylogeny is then encoded as a graph and analyzed using the STEPHY framework to perform regression tasks and a classification task (Fig. 1b–c). The regression targets include the basic reproduction number, $R_{0i}$; the recovery rate, $\gamma_{i}$; and the source-sink scores, $SSS_{i}$. Whereas $R_{0i}$ and $\gamma_{i}$ are model parameters that directly govern outbreak dynamics, $SSS_{i}$ is a derived measure of each region’s role in exporting or receiving infections in the realized outbreak (65, 66). The score ranges from (−1) to (+1), with (+1) indicating a pure source, (−1) a pure sink, and intermediate values indicating net source or sink roles. These estimates can help prioritize regions for targeted surveillance and control. Using simulations, we observe that population size, migration rates, and transmission intensity can each shape $SSS_{i}$ under different simulation scenarios (**Supplementary Fig. 1–3, Supplementary Table 1–3**). The classification target is the index location, defined as the geographic region where the simulated outbreak began. In the resulting phylogeny, this corresponds to the ancestral geographic state assigned to the root. Unlike the continuous epidemiological quantities predicted through regression, index-location inference is formulated as a binary node-classification task, in which each region is labeled as the index location or not. Accordingly, STEPHY uses mean squared error (MSE) loss for the continuous regression targets and cross-entropy (CE) loss for index-location classification.

To evaluate STEPHY inference in a complex structured system, we simulate outbreaks across 12 interconnected populations with heterogeneous population sizes and epidemiological parameters. This setting represents a complex structured system that is computationally challenging for conventional Bayesian phylogeographic methods. For each region i, population size is sampled between 5,000 to 50,000 individuals, $R_{0i}$ from 2 to 8, and $\gamma_{i}$ from 0.05 to 0.25. Each outbreak was simulated for a duration corresponding to 6–18 mean infectious periods, with phylogenies limited to 5,000 sampled tips. Among 100,000 simulated outbreaks, 89,980 phylogenies are retained after quality filtering. Tree sizes range from 561 to 5,000 tips, with a mean of 3,190.5 tips. Region-specific subtree sizes range from 31 to 702 tips, with a mean of 265.9 tips. Full simulation settings and parameter ranges are provided in **Supplementary Table 1**.

### STEPHY Enables Accurate and Rapid Inference in a Complex Metapopulation System

STEPHY supports both point-estimate and uncertainty-aware inference. Point-estimate inference returns a single prediction for each target, including continuous parameter estimates and the most likely index location. For uncertainty-aware inference, STEPHY implements conformal prediction. Regression targets are calibrated with conformalized quantile regression (CQR) to generate prediction intervals (71), while index-location predictions are calibrated with regularized adaptive prediction sets (RAPS) to generate prediction sets containing one or more plausible index locations (72). Both procedures use a held-out calibration set and are configured for 95% marginal coverage. The results below focus on these conformal-prediction outputs.

STEPHY achieves strong performance on both regression and classification tasks (Fig. 2a). On the held-out test set, median quantile predictions of $R_{0i},\gamma_{i}$, and $SSS_{i}$ achieve $R^{2}$ values of 0.9236, 0.9795, and 0.8585, respectively, while point predictions identify the index location with an accuracy of 0.9417. CQR achieves empirical coverages of 0.9512, 0.9515, and 0.9517 for $R_{0i},\gamma_{i}$, and $SSS_{i}$, respectively, while maintaining mean prediction-interval widths of 1.34, 0.028, and 0.34. RAPS achieves conservative coverage of 0.9945, with a mean prediction-set size of 3.21. STEPHY also performs inference in milliseconds per phylogeny (Table 1). Across 5,999 test trees per task, mean per-tree inference times are 6.58 ms for $R_{0i}$, 6.54 ms for $\gamma_{i}$, 6.55 ms for $SSS_{i}$, and 6.71 ms for index-location classification. These measurements are obtained on a single Intel Xeon Gold 6242 CPU core at 2.80 GHz using PyTorch 2.4 and DGL 2.4 with 32-bit floating-point precision, a batch size of 1, and single-threaded execution. Inference time remains nearly independent of phylogeny size, with Pearson correlations between forward-pass time and tree size close to zero for all tasks ($R_{0i}:r=-0.009;\gamma_{i}:r=-0.001;SSS_{i}:r=0.033$; index location: r=0.020). This near size-independent runtime reflects STEPHY’s fixed-shape input encoding.

To assess whether modeling dependencies among regional outbreaks improves inference, we performed an ablation analysis in which the graph component of STEPHY was removed (Fig. 2b **and Supplementary Fig. 4**). We refer to this location-independent baseline as CBLV-CNN. It retains the same per-region subtree inputs, represented using compact bijective ladderized vectors (CBLVs), as well as the same convolutional encoders and prediction heads as STEPHY, but excludes the region-level graph representation, DTW-derived edge features, and message-passing layer. Each region is therefore analyzed independently, whereas STEPHY integrates information across regions through edge-aware message passing. STEPHY substantially improves regression inference relative to CBLV-CNN, increasing $R^{2}$ by 0.1473 for $R_{0i}$, 0.0761 for $\gamma_{i}$, and 0.1438 for $SSS_{i}$, and decreasing mean prediction-interval widths by 40.4%, 52.5%, and 27.7%, respectively, at comparable coverage. In contrast, the two models perform similarly in index-location classification, with nearly identical accuracy and prediction-set sizes.

### STEPHY Ranks Transmission Hotspots and Generalizes Across Population Scales

For STEPHY’s regression tasks, the model is trained to minimize prediction error for node-level targets. However, a key practical question is whether these predictions can also correctly identify regions with the highest transmission potential. Using $R_{0i}$ as an example, STEPHY correctly identified the region with the highest $R_{0i}$ in 47.8% of held-out test simulations, compared with 32.0% for CBLV-CNN (Fig. 3a). Exact identification is intrinsically challenging because true $R_{0i}$ values are often closely spaced, with a median difference of only 0.11 between adjacent ranked regions (Fig. 3b). When one mismatch was allowed in identifying the top-k regions, STEPHY achieved accuracies ranging from 90.9% to 84.3% for k=2 to 5, substantially outperforming CBLV-CNN (79.0% to 60.5%). Results for identifying regions with high $SSS_{i}$ values are shown in **Supplementary Fig. 5**.

We next evaluated whether STEPHY trained on smaller simulated populations can generalize to larger populations (Fig. 3c–f, **Supplementary Fig. 6–7**). This analysis addresses a practical consideration of simulation-based inference: generating large training datasets becomes increasingly computationally expensive as population size increases. Models trained on outbreaks with regional population sizes ranging from 5,000 to 50,000 individuals were therefore evaluated on outbreaks in which all population sizes were scaled by factors of 1, 2, and 3, while sampling rates were reduced proportionally to maintain comparable phylogeny sizes. STEPHY remains largely robust as population sizes extend beyond the training distribution, although performance gradually declines with increasing scale. Across all three regression targets, STEPHY consistently outperforms CBLV-CNN. At the threefold population scale, STEPHY achieves higher $R^{2}$ values than CBLV-CNN for $R_{0i}$ (0.799 versus 0.739), $\gamma_{i}$ (0.851 versus 0.801), and $SSS_{i}$ (0.843 versus 0.625). Among the three regression targets, $SSS_{i}$ remains the most robust to increasing population scale. STEPHY’s $R^{2}$ for $SSS_{i}$ decreases only slightly, from 0.867 to 0.843, whereas that of CBLV-CNN declines more substantially, from 0.745 to 0.625. Index-location classification also remains stable for both models, with accuracy changing from 0.940 to 0.931 for STEPHY and from 0.944 to 0.927 for CBLV-CNN.

### Preparing Densely Sampled Danish SARS-CoV-2 Phylogenies

We focus our real-world application on Denmark, which has one of the highest SARS-CoV-2 sequencing efforts worldwide, supported by extensive testing, genomic surveillance, and demographic record-keeping (18). In Denmark, sequencing coverage consistently exceeds 60% of PCR-positive cases and reaches up to 80% between March and November 2021, providing unusually comprehensive representation of the national epidemic (67). This dense genomic surveillance provides a unique opportunity to use STEPHY to trace how the epidemiological roles of different Danish regions change across successive waves of the pandemic.

To prepare the empirical genomic dataset, we first estimate weekly detected cases for each variant and region by combining regional case counts reported by Statens Serum Institut (SSI) with variant proportions derived from GISAID sequences (Fig. 4a). We then compare these estimates with the corresponding numbers of available sequences (Fig. 4b) to quantify weekly sequencing proportions before subsampling (Fig. 4c). Subsampling is necessary because Denmark’s genomic surveillance contains nearly 300,000 SARS-CoV-2 genomes, making direct phylogenetic reconstruction computationally impractical. In addition, STEPHY approximates regional epidemic trajectories from the density of tip sampling times, which requires broadly consistent sampling proportions across regions within each variant period. To satisfy these requirements, we cap each variant-region-week stratum at a variant-specific baseline sampling proportion: 8% for Alpha during weeks 01–22, 12% for Delta during weeks 23–35, and 4% for Omicron during weeks 47–52. After subsampling, sequencing coverage is more consistent within each variant period (Fig. 4d).

Following proportional subsampling, sequences are first quality filtered using Nextclade (73), yielding three datasets corresponding to the Alpha (n=6,216), Delta (n=6,581), and Omicron (n=8,514) variant periods. For each dataset, we infer one maximum-likelihood tree and 40 standard nonparametric bootstrap replicates using IQ-TREE (74) under a GTR model. All 41 trees per variant period are then time-calibrated under identical settings. Each sequence is assigned to a target lineage using its Nextclade clade label, and the corresponding lineage is identified within each tree as the most recent common ancestor (MRCA) of its assigned tips. This approach yields five target lineages (Fig. 5a): Alpha 20I (n=6,215), Delta 21I (n=571) and 21J (n=5,856), and Omicron 21K (n=7,177) and 21L (n=1,336).

Time to the most recent common ancestor (TMRCA) estimates are consistent with the known emergence of each lineage and fall within the full bootstrap range in every case. Alpha 20I dates to December 5, 2020, with a bootstrap range from July 25 to December 24, 2020. Delta 21I and 21J date to May 22 and May 16, 2021, respectively, with bootstrap ranges from April 25 to May 28, 2021, and from March 17 to May 27, 2021. Omicron 21L dates to November 27, 2021, with a bootstrap range from November 24 to December 10, 2021. The TMRCA for Omicron 21K, November 13, 2021, coincides with the upper limit of its bootstrap range. This wide range indicates greater topological uncertainty near the base of this large, rapidly expanding lineage (**Supplementary Fig. 8**).

### STEPHY Resolves Regional Epidemiological Dynamics Across Danish SARS-CoV-2 Waves

We trained a Denmark-specific STEPHY model on 100,000 simulated outbreaks across five interconnected populations corresponding to Hovedstaden, Midtjylland, Syddanmark, Sjælland, and Nordjylland, with fixed population sizes of 1.86 million, 1.33 million, 1.22 million, 0.84 million, and 0.59 million individuals, respectively. The simulations spanned broad epidemic conditions, with $R_{0i}$ ranging from 0.5 to 4, $\gamma_{i}$ from 0.07 to 0.23, outbreak durations from 20 to 240 days, and phylogenies containing up to 8,000 sampled tips. Using STEPHY’s point-estimate mode (**Supplementary Fig. 9**), we applied the trained model to five reconstructed phylogenies representing major Danish SARS-CoV-2 lineages, reporting a single estimate for each regional epidemiological quantity and the most likely index location for each lineage. These lineages arose under markedly different epidemiological conditions, including variation in population immunity, vaccination coverage, public health interventions, sampling intensity, and epidemic scale, providing a challenging real-world application of STEPHY. Despite these differences, inference remained rapid, requiring approximately 19 ms per-tree on average under the same hardware and software settings (Table 2). Full simulation settings and parameter ranges are provided in **Supplementary Table 2**.

Across the five Danish lineages, Hovedstaden, the Copenhagen capital region, consistently has the highest inferred $SSS_{i}(20\text{I}:+0.35;21\text{I}:+0.48;21\text{J}:+0.31;21\text{K}:+0.59;21\text{L}:+0.81)$, indicating that it acted as the primary source of viral dissemination to other Danish regions. Because Hovedstaden is also Denmark’s most populous region, we examined whether high $SSS_{i}$ simply reflects population size. Held-out simulations indicate that it does not (**Supplementary Fig. 2**). Across test simulations, the Spearman rank correlation between $SSS_{i}$ rank and population-size rank was close to zero (meanρ=0.031), with positive correlations in only 50.4% of outbreaks. The spread of $SSS_{i}$ values across regions (max(SSS)-min(SSS)) also increases substantially over the course of the pandemic, from 0.6 in Alpha 20I to 1.1 in Delta 21J and 1.5 in Omicron 21L. This pattern indicates increasingly pronounced source-sink differentiation among Danish regions, with the strongest spatial heterogeneity observed during the Omicron waves (Fig. 5b–f). Estimated $R_{0i}$ values increase across the major SARS-CoV-2 variant waves in Denmark. Averaged across the five Danish regions, the mean inferred $R_{0i}$ rose from 1.10 for Alpha 20I to 1.17 and 1.14 for the Delta 21J and 21I, and further to 1.34 and 2.23 for the Omicron 21K and 21L lineages (Fig. 5g–k).

The inferred patterns are broadly stable to phylogenetic uncertainty. For most lineage-region estimates, predictions from the maximum-likelihood trees fall within the full range obtained from the 40 bootstrap trees (46 of 50 estimates; 24 of 25 for $SSS_{i}$ and 22 of 25 for $R_{0i}$). Bootstrap variation differs by target and lineage. It was generally wider for $SSS_{i}$ than for $R_{0i}$, and Omicron 21L exhibits the widest bootstrap variation among the five lineages (Fig. 5b–k).

## Discussion

Translating large-scale genomic surveillance data into actionable regional epidemic intelligence remains a challenge for most public health systems due to the computational bottlenecks of likelihood-based phylogeographic methods (29, 36, 38, 75). Simulation-based deep learning approaches (44, 46–49, 76, 77) offer an alternative through amortized inference: rather than repeatedly evaluating a likelihood for each new dataset, the computational cost is paid during training on simulated outbreaks with known epidemiological parameters, after which inference on a new phylogeny requires only a single forward pass. Here, we present STEPHY, a pathogen-agnostic graph neural inference framework for rapid, region-specific epidemic estimation from large time-calibrated phylogenies. STEPHY introduces a graph representation of viral phylogenies that encodes regional subtree structure as nodes and temporal epidemic synchronization as directed edges, enabling joint inference of region-specific transmission dynamics through edge-aware message passing. STEPHY can be adapted to different pathogens, population structures, and surveillance settings by modifying the simulator and training distribution. Once trained, STEPHY analyzes phylogenies containing thousands of sequences in milliseconds, making structured phylogeographic inference practical for timely genomic surveillance and epidemic response.

STEPHY accurately estimates three continuous epidemiological quantities: the regional reproduction number, recovery rate, and source-sink score. Across all three targets, STEPHY outperforms an otherwise matched ablation model that analyzes each regional subtree independently, achieving higher predictive accuracy and narrower prediction intervals at comparable coverage. The ablation results therefore show that STEPHY’s design successfully captures cross-regional signals that are missed when regional outbreaks are analyzed in isolation. By contrast, STEPHY provides only limited improvement in index-location classification, with both models achieving similar accuracy and prediction-set sizes.

Our application to Denmark illustrates both the empirical and operational value of STEPHY for genomic surveillance. Empirically, STEPHY recovered reproduction-number estimates broadly consistent with those from previous analyses of Danish SARS-CoV-2 data (18, 44), while extending to spatially explicit inference of region-specific epidemic dynamics and source-sink roles. STEPHY also accommodated substantially larger phylogenies, including one lineage with more than 7,000 sequences. Across five major lineages spanning the Alpha, Delta, and Omicron waves, STEPHY consistently identified Hovedstaden as the primary source of viral dissemination within Denmark. Hovedstaden, the capital region, includes Copenhagen, the country’s largest urban center and a major transportation hub. Simulation analyses show that this dominant source role is not simply a consequence of Hovedstaden’s larger population, pointing instead to the importance of regional connectivity and its position within the national transmission network. We also observed increasingly pronounced source-sink differentiation across successive variant waves, with the strongest regional heterogeneity during Omicron. Operationally, STEPHY accepts time-calibrated phylogenies as input and can therefore integrate with established genomic surveillance pipelines such as Nextstrain (78). Indeed, in the Denmark analysis, sequence processing and time-tree reconstruction used components of the Nextstrain workflow, demonstrating how STEPHY can serve as a downstream inference layer that rapidly translates reconstructed phylogenies into region-specific epidemiological estimates.

An important feature of STEPHY is its robustness to phylogenetic uncertainty. Regional estimates inferred from maximum-likelihood trees were generally consistent with those obtained from bootstrap replicates, indicating that the framework does not rely heavily on a single tree topology. This robustness is particularly valuable for genomic surveillance, where large phylogenies often contain considerable topological uncertainty arising from limited sequence divergence and uneven sampling.

Several limitations should be considered. First, as with all simulation-based approaches, STEPHY can only learn relationships represented in its training data. Predictions may become biased if real outbreaks differ substantially from the simulated scenarios used for training. Developing simulation strategies that better reflect specific epidemiological systems therefore remains essential for successful application. Second, our current implementation assumes a fully connected migration structure and summarizes relationships between regions solely through temporal synchronization patterns. Future work could incorporate external information, such as human mobility, transportation networks, or geographic distance, to construct more informative graph structures and edge features.

Third, our simulation framework assumes that tip density is proportional to prevalence, whereas the Danish application more closely approximates incidence-based sampling. Although these two quantities are closely related when infectious-period distributions are similar across regions, this mismatch may become important in systems with substantial spatial variation in recovery dynamics or sampling practices. Future work could address this limitation by explicitly modeling heterogeneous sampling processes during simulation and training.

Finally, the current framework focuses on inferring static region-level quantities from a single phylogeny. Many public health questions require understanding how transmission dynamics change over time. Extending STEPHY to infer time-varying epidemiological parameters, accommodate changing migration patterns, and integrate additional data streams represents an important direction for future research.

In summary, STEPHY provides a scalable and readily deployable phylogeographic toolkit that rapidly infers regional epidemic dynamics directly from large phylogenies. The accompanying documented toolkit supports outbreak simulation, model training, uncertainty quantification, and inference, allowing it to be adapted to different epidemiological settings. Together, these capabilities provide a practical foundation for rapid phylogeographic analysis and timely, targeted public health decision-making.

## Materials and Methods

### Stochastic SIR Metapopulation Model

STEPHY is trained on simulated outbreaks for which the underlying epidemiological quantities are known. Although the framework is agnostic to the choice of epidemic simulator, we use a stochastic SIR metapopulation model in this study as a simple and general benchmark. The model is defined over n discrete regions. For each region $i,S_{i},I_{i},R_{i}$, and ${Smp}_{i}$ denote the numbers of susceptible, infectious, recovered, and sampled individuals, respectively.

The model includes four stochastic event types: infection, migration, recovery, and sampling. Each event is represented as a transition between epidemiological compartments, with the compartments on the left denoting the state before the event and those on the right denoting the state after the event.

The quantity above each arrow specifies the corresponding event rate. Infection depends on interactions between susceptible and infectious individuals, producing a total event rate of $\beta_{i}S_{i}I_{i}/N_{i}$, where $\beta_{i}$ is the transmission rate and $N_{i}$ the population size of region i. Migration, recovery, and sampling each act independently on infectious individuals, with per-individual rates $m_{ij},\gamma_{i}$, and δ respectively. The resulting transmission history is then converted into a time-calibrated phylogeny, with sampled infections forming the tips. The four events are:

(1) $$S_{i}+I_{i}\overset{\frac{\beta_{i}}{N_{i}}}{\to }2I_{i}\text{(infection)}$$

(2) $$I_{i}\overset{m_{ij}\;}{\to }I_{j}\text{(migration)}$$

(3) $$I_{i}\overset{\gamma_{i}\;}{\to }R_{i}\text{(recovery)}$$

(4) $$I_{i}\overset{\delta \;}{\to }R_{i}+Smp_{i}\text{(sampling)}$$

We generate outbreak trajectories using the Gillespie stochastic simulation algorithm (62, 70), which repeatedly draws the time to the next event and its type in proportion to the event rates above. The expected behavior of the stochastic process is described by the following system of ordinary differential equations:

(5) $$\frac{dS_{i}}{dt}=-\frac{\beta_{i}}{N_{i}}S_{i}I_{i}$$

(6) $$\frac{dI_{i}}{dt}=\frac{\beta_{i}}{N_{i}}S_{i}I_{i}+\sum_{j\neq i}^{n}m_{ji}I_{j}-\sum_{j\neq i}^{n}m_{ij}I_{i}-\left(\gamma_{i}+\delta_{i}\right)I_{i}$$

(7) $$\frac{dR_{i}}{dt}=\left(\gamma_{i}+\delta \right)I_{i}$$

(8) $$\frac{dSmp_{i}}{dt}=\delta I_{i}$$

### Simplifications for Computational Tractability

We adopt the following simplifying assumptions, which specify the model and make outbreak simulation under it tractable.

(1) Only infected individuals migrate. Susceptible and recovered individuals remain in their regions of origin. Thus, migration redistributes infection across regions without altering the susceptible pool available for local transmission. (2) Sampling immediately moves an individual from the infectious compartment to the recovered compartment. Sampled individuals are treated as no longer infectious and cannot transmit or migrate after sampling. (3) The population size of each region, $N_{i}$, is held fixed at its initial value, $N_{i}=S_{i}(0)+I_{i}(0)+R_{i}(0)$, throughout the simulation. (4) Rather than specifying $\beta_{i}$ directly, we derive it through a region-specific basic reproduction number $R_{0i}$, which we treat as the intrinsic transmission potential within region i, independent of migration:

(9) $$R_{0i}=\frac{\beta_{i}}{\gamma_{i}+\delta }$$

Rearranging and substituting the constant population size yields the per-combination rate for Equation 1:

(10) $$\frac{\beta_{i}}{N_{i}}=\frac{R_{0i}\left(\gamma_{i}+\delta \right)}{S_{i}(0)+I_{i}(0)+R_{i}(0)}$$

### Parameter Settings for Simulated Datasets

Each outbreak simulation is configured by the following parameters: (1) the number of regions n; (2) the initial compartment sizes $S_{i}(0),I_{i}(0)$, and $R_{i}(0)$ for each region, which also fix the constant population sizes $N_{i}$; (3) the region-specific epidemic parameters $R_{0i},m_{ij},\gamma_{i}$, and δ; and (4) a stopping condition, such as a maximum simulation time or a target tree size. Once these inputs are set, we simulate an outbreak and its phylogeny using the Gillespie algorithm described above.

Training and evaluating STEPHY requires not a single phylogeny but a dataset of many phylogenies that together span a broad range of spatial epidemic scenarios. We assemble such a dataset by repeatedly sampling parameter configurations from specified distributions. Varying these parameters and their distributions lets the same framework generate very different datasets, making the simulator highly adaptive. Full parameter settings are provided in the supplementary materials: the simulation benchmark in **Supplementary Table 1**, the Denmark inference dataset in **Supplementary Table 2**, and the remaining datasets used for training and evaluation in **Supplementary Table 3–6**.

### Graph Construction

Graph neural networks (GNNs) extend neural networks to data represented as graphs, where entities are encoded as nodes and their relationships as edges (79). Through message passing, each node updates its representation using information from connected nodes and edges, allowing the model to learn both region-specific patterns and dependencies among regions (80).

STEPHY introduces a new graph representation tailored to geographically structured phylogenies. Each time-calibrated, bifurcating phylogeny is mapped to a fully connected directed graph whose nodes represent distinct regions. For n regions, we add one directed edge for every ordered pair (i,j) with i≠j, resulting in n(n-1) edges and no self-loops.

Node and edge features are then assigned to the graph. Node features summarize each region’s subtree, the clade containing its sampled tips. Edge features describe the temporal relationship between each pair of region-specific outbreaks, using sampling-time profiles as a proxy for epidemic dynamics. These two feature types are constructed separately, as described below.

### Node Features

The Compact Bijective Ladderized Vector (CBLV) is a bijective encoding of the subtree (44, 47, 81). We compute it using a ladderized in-order traversal. At each branch point, we first descend into the deeper child first, then record the node itself, then finally descend into the other child. This traversal produces an m×4 CBLV matrix, where m is the number of sampled tips. The CBLV matrix is organized by node type: columns 0 and 2 describe the tips, while columns 1 and 3 describe the internal nodes. Column 0 gives each tip’s depth relative to the internal node in the same row (column 1), that is, how far the tip lies below that node; column 1 gives each internal node’s absolute depth, measured from the whole-tree root to the node; column 2 gives each tip’s branch length to its parent; and column 3 gives each internal node’s branch length to its parent. Because internal-node depths are measured from the whole-tree root rather than from the subtree’s own most recent common ancestor (MRCA), each subtree is positioned within the full phylogeny rather than encoded in isolation. All entries are then normalized by the whole-tree height, placing them roughly in the range [0,1]. Because subtrees vary in size, m differs across regions. We therefore standardize the number of rows to a fixed value, W, defined as the largest per-region tip count in the dataset. Each CBLV matrix is zero-padded along the row axis to a fixed size of W×4.

The fixed-size W×4 matrix is passed to a 3-branch 1-D convolutional encoder (82) that convolves along the row axis and treats the four columns as input channels, with each branch capturing structure at a complementary scale. The plain branch applies three convolutions with 12, 24, and 48 filters (kernels 3, 5, 7; stride 1; ReLU) to capture fine local detail. The strided branch applies two convolutions with 12 and 24 filters (kernels 7, 9; strides 3, 6; ReLU) to downsample the matrix into a coarse summary. The dilated branch (83) applies two convolutions with 12 and 24 filters (kernels 3, 5; dilations 3, 5; ReLU) to capture sparse long-range structure. Each branch ends with adaptive average pooling, which collapses the row axis to one value per filter and yields fixed-length branch embeddings of 48, 24, and 24 dimensions regardless of W. Concatenating them forms a 96-dimensional CBLV embedding vector.

Each subtree is also represented by a 5-dimensional summary-statistic vector: the MRCA’s root distance, which anchors the subtree in the global frame; the earliest and latest tip times; the mean MRCA-to-tip distance; and the tip count. These statistics are log-transformed and then z-scored using training-set statistics only. The normalized summary-statistic vector is then passed through a two-layer MLP (5 → 64 → 32, ReLU) to produce a 32-dimensional summary-statistic embedding vector. Concatenating this vector with the 96-dimensional CBLV embedding vector gives the final 128-dimensional node-feature vector, denoted $h_{i}$ for region i (Fig. 1b).

### Edge Features

Edge features describe the temporal synchronization between outbreaks in pairs of regions. For each region, we convert the sampling times of its tips into a smooth sampling-intensity curve using a Gaussian kernel density estimate. For every directed pair, we align the two curves using dynamic time warping (DTW) with a squared-error cost, yielding two quantities (63). The first is the DTW distance, defined as the accumulated cost along the optimal path, which measures dissimilarity in the shape of the two sampling histories. The second is the set of offsets along the optimal path. The mean and standard deviation of these offsets capture how far one region’s sampling leads or lags the other, and how consistent that lag is. Each directed edge j→i therefore carries a 3-dimensional edge-feature vector, denoted as $e_{j\to i}$ (Fig. 1b). The DTW distance and lag standard deviation are log-transformed and then z-scored, while the mean lag is z-scored directly. All transformations use training-set statistics only.

In summary, each graph with n nodes is described by n node-feature vectors $h_{i}\in R^{128}$ and n(n-1) edge-feature vectors $e_{j\to i}\in R^{3}$.

### Message Passing with Edge-Aware Attention

Using the node-feature vectors $h_{i}$ and edge-feature vectors $e_{j\to i}$ defined above, STEPHY performs a single round of message passing over the fully connected directed graph. Each edge feature captures the temporal relationship between a pair of regional outbreaks, allowing the model to learn which regions provide the most relevant context for each target region.

For an incoming edge from region j to region i, a two-layer MLP (3 → 6 → 1, ReLU) maps the edge-feature vector to a scalar attention score:

(11) $$s_{j\to i}=MLP\left(e_{j\to i}\right).$$

Because the edge features are directional, $s_{j\to i}$ and $s_{i\to j}$ are estimated independently and need not be equal. For each target region i, the scores of all incoming edges are normalized using a softmax:

(12) $$\alpha_{j\to i}=\frac{\text{exp}\left(s_{j\to i}\right)}{\sum_{k\neq i}\text{exp}\left(s_{k\to i}\right)}.$$

The resulting attention weights satisfy $\sum_{j\neq i}\alpha_{j\to i}=1$ and quantify the relative contribution of each neighboring region to the representation of region i. The neighboring node representations are then combined as a weighted sum:

(13) $$agg_{i}=\sum_{k\neq i}\alpha_{k\to i}\;h_{k}.$$

This operation produces a 128-dimensional aggregated-neighbor vector that summarizes the rest of the phylogeny from the perspective of region i, with greater weight assigned to regions whose outbreaks are more temporally informative. Concatenating this vector with the region’s own node-feature vector yields a 256-dimensional representation that contains both the region-specific subtree signal and its cross-regional context:

(14) $$z_{i}=h_{i}\|agg_{i},$$

where || denotes vector concatenation. Each regional representation $z_{i}$ is then decoded independently by a four-layer MLP head (256 → 128 → 64 → 32 → output, ReLU; Fig. 1c).

To assess whether STEPHY’s graph-based joint modeling of regional outbreaks improves over independent region-by-region analysis, we compare it against CBLV-CNN, an ablation model that retains the same subtree encoders and prediction heads but removes the graph representation, edge features, and message-passing layer. CBLV-CNN uses the same CBLV encoder and summary-statistic branch as STEPHY but expands them to a 192-dimensional CBLV embedding and a 64-dimensional summary-statistic embedding, respectively. Their concatenation produces a 256-dimensional representation for each region, matching the dimensionality of the representation used by STEPHY after concatenating $h_{i}$ and ${\text{agg}}_{i}$. Both models then use the same four-layer prediction head. Therefore, differences in performance reflect the contribution of message passing rather than differences in representation size or model capacity.

### Regression and Classification Tasks

STEPHY is trained as one model per task across four prediction tasks, split into regression and classification by target quantity. The three regression tasks estimate one continuous value per region: the basic reproduction number, $R_{0i}$; the recovery rate, $\gamma_{i}$; and the source-sink score, $SSS_{i}\overset{ˆ}{\text{I}}[-1,+1]$, where values near +1 indicating a net viral source and values near −1 indicating a net viral sink. Unlike $R_{0i}$ and $\gamma_{i}$, which directly govern outbreak dynamics, $SSS_{i}$ is derived from the realized movement of infection between regions.

For a regression task, the prediction head produces one scalar estimate ${\overset{ˆ}{y}}_{g,i}$ for region i in tree g. The model is trained by minimizing the mean squared error across all G trees and N regions:

(15) $${ℒ}_{\text{MSE}}=\frac{1}{GN}\sum_{g=1}^{G}\sum_{i=1}^{N}{\left({\overset{ˆ}{y}}_{g,i}-y_{g,i}\right)}^{2}.$$

The target values are standardized using the mean and standard deviation of the training set. Predictions are transformed back to their original scale for evaluation and reporting. Training stops early when the validation MSE no longer improves.

The classification task identifies the index location. For each tree g, the prediction head produces one logit $z_{g,i}$ for every candidate region i. These logits are converted into predicted probabilities $p_{g,i}$ across the N regions using the softmax function. The model is trained by minimizing the categorical cross-entropy loss:

(16) $${ℒ}_{\text{CE}}=-\frac{1}{G}\sum_{g=1}^{G}\text{log}\;p_{g,c_{g}},$$

where $c_{g}$ denotes the true index location for tree g, and $p_{g,c_{g}}$ is the predicted probability assigned to that location. Each tree contributes one classification outcome, and training stops early based on validation accuracy.

### Point Estimate and Conformal Prediction

By default, each task produces a point prediction: one value per region for regression or one predicted region for classification. To quantify uncertainty, we optionally apply conformal prediction using a held-out calibration set, targeting coverage 1-α, with α=0.05 by default. For regression, we use conformalized quantile regression (CQR) (71). For each input x, the prediction head outputs three quantile estimates: a lower quantile ${\overset{ˆ}{q}}_{\text{lo}}(x)$ at level α/2, a median ${\overset{ˆ}{q}}_{\text{mid}}(x)$ at level 0.5, and an upper quantile ${\overset{ˆ}{q}}_{\text{hi}}(x)$ at level 1-α/2. More generally, ${\overset{ˆ}{q}}_{\tau }(x)$ denotes the model-predicted τ-quantile of the target for input x. These quantiles are trained using the pinball loss:

(17) $$\rho_{\tau }(u)=\text{max}\{\tau u,(\tau -1)u\},$$

where $u=y-{\overset{ˆ}{q}}_{\tau }(x)$ is the residual between the observed target y and the predicted τ-quantile. For each calibration observation $\left(x_{i},y_{i}\right)$, the conformity score measures how far the observed value falls outside the predicted lower and upper quantiles:

(18) $$E_{i}=\text{max}\left\{{\overset{ˆ}{q}}_{\text{lo}}\left(x_{i}\right)-y_{i},y_{i}-{\overset{ˆ}{q}}_{\text{hi}}\left(x_{i}\right)\right\}.$$

The empirical 1-α quantile of these scores defines a correction $\overset{ˆ}{q}$, giving the calibrated interval $\left[{\overset{ˆ}{q}}_{\text{lo}}(x)-\overset{ˆ}{q},{\overset{ˆ}{q}}_{\text{hi}}(x)+\overset{ˆ}{q}\right]$. The median prediction ${\overset{ˆ}{q}}_{\text{mid}}(x)$ is used as the point estimate.

For classification, we use regularized adaptive prediction sets (RAPS) after standard cross-entropy training (72). For a given tree, regions are ordered by decreasing softmax probability, $p_{\left(1\right)}\geq p_{\left(2\right)}\geq \cdots \geq p_{\left(N\right)}$, where $p_{\left(k\right)}$ denotes the k-th largest probability. For a calibration tree whose true region has rank r in this ordering, the conformity score sums the probability mass down to the true region and adds a rank penalty:

(19) $$E=\sum_{k=1}^{r}p_{\left(k\right)}+\lambda \;\text{max}\left(r-k_{\text{reg}},0\right).$$

Here, λ=0.01 controls the penalty strength, and $k_{\text{reg}}=2$ specifies the number of top-ranked regions exempt from the penalty. The empirical 1-α quantile of the calibration scores defines the prediction-set threshold. At inference, regions are added in decreasing probability order until this threshold is reached, producing a single-region prediction when confidence is high and a larger set when the prediction is ambiguous.

### Application to the 2021 SARS-CoV-2 epidemic in Denmark

We applied STEPHY to a real surveillance setting, the SARS-CoV-2 epidemic in Denmark during 2021. Denmark is well suited to this test. Its comprehensive sequencing programme produced one of the densest genomic records of any national epidemic, and the year 2021 captured three successive variant waves, Alpha, Delta, and Omicron, unfolding across five administrative regions of contrasting size and connectivity. To assemble the input for STEPHY, we integrated two complementary data sources, national case surveillance and viral genomes, and subsampled the genomes to a controlled and regionally comparable rate, as described below.

### Surveillance data

We obtained daily confirmed case records from Statens Serum Institut (SSI), reported per day and per region (18). Records were aggregated into ISO epiweeks spanning 4 January 2021 to 2 January 2022. This yielded, for each epiweek, a per region confirmed case count that served as the denominator for all subsequent sampling calculations.

### Genome data

Genomes were downloaded from the GISAID database (84) and filtered to the same population and period. Each sequence was assigned to Alpha, Delta, or Omicron from its GISAID variant annotation. Because the surveillance counts are not variant resolved, we estimated variant specific detected cases by multiplying each region week’s confirmed cases by the proportion of that week’s sequences belonging to each variant, using the surveillance data for the magnitude of detection and the genomes for its variant composition.

### Proportional subsampling

To prevent regions with more intensive sequencing from disproportionately influencing the phylogenies, we subsampled genomes so that each region was represented at an approximately equal rate relative to its estimated number of detected cases. Subsampling was conducted within variant-specific epidemiological-week windows using fixed target rates: 8% for Alpha (2021-W01 to W22), 12% for Delta (W23 to W35), and 4% for Omicron (W47 to W52). Within each stratum defined by variant, epidemiological week, and region, the target number of genomes was calculated as the floor of the target sampling rate multiplied by the estimated number of detected cases, with a minimum of one genome retained for each nonempty stratum. Strata containing more genomes than the target were randomly down sampled without replacement, whereas all genomes were retained from strata at or below the target. This procedure yielded 6,372 Alpha, 6,595 Delta, and 8,571 Omicron genomes, for a total of 21,538 genomes. Each genome was annotated with its accession number, sampling date, epidemiological week, Pango lineage, variant, and region. Comparison of sampling proportions before and after subsampling confirmed that the substantial regional variation in the raw data was reduced to a more consistent sampling rate across regions (Fig. 4).

### Alignment and quality filtering

We constructed the alignment using the Nextstrain pipeline (78) and applied two stages of quality control. The first stage, implemented as part of the Nextstrain pipeline, removed sequences shorter than 27 kb, sequences included on a manual exclusion list, and molecular-clock outliers whose divergence was inconsistent with their sampling dates. The second stage removed genomes with evidence of sequencing or assembly errors, specifically those assigned an overall “bad” quality rating by Nextclade (73) because of excessive private mutations, frameshifts, mixed sites, stop codons, clustered SNPs, or an aggregate quality score exceeding the specified threshold. Together, the two stages removed 230 genomes, leaving 6,215 Alpha, 6,580 Delta, and 8,513 Omicron sequences, for a total of 21,308 sequences used in phylogenetic inference.

### Maximum-likelihood tree and bootstrap replicates

For each of the three variants, we inferred a maximum-likelihood phylogeny from the masked alignment using IQ-TREE (74), with a GTR substitution model, and ten parsimony starting trees. We also generated 40 standard nonparametric bootstrap replicates per variant, each based on an independent tree search using a site-resampled alignment. This procedure produced one maximum-likelihood tree and 40 bootstrap trees per variant, yielding 123 trees in total. Although we reconstructed only three variant-level phylogenies, they collectively contained the five target Nextstrain lineages: 20I within Alpha; 21I and 21J within Delta; and 21K and 21L within Omicron. Lineage membership was determined using Nextclade’s per-genome labels, which are assigned from sequence data alone and are therefore independent of tree topology. For each lineage, we identified the most recent common ancestor of all tips assigned to that lineage in every tree and extracted the corresponding subtree. Downstream inference was therefore performed separately for each lineage rather than on the complete variant-level phylogeny.

### Time-scaled trees

Because STEPHY requires branch lengths measured in time, we dated all 41 topologies per variant—the maximum-likelihood tree and 40 bootstrap replicates—using TreeTime through augur refine (78, 85). We fixed the molecular clock rate at 8 × 10^−4^ substitutions per site per year, with a standard deviation of 4 × 10^−4^, used an optimized coalescent prior, inferred node dates marginally with confidence intervals, and resolved polytomies stochastically. We then converted each tree to time units by setting each branch length to the difference between the inferred dates of its parent and child nodes. Identical refinement parameters were applied to the maximum-likelihood tree and all bootstrap replicates, so variation across estimates primarily reflected topological uncertainty.

## Supplementary Material

Supplementary Files

This is a list of supplementary files associated with this preprint. Click to download.

- 2026LyustephySI.pdf

## Figures and Tables

**Figure 1.**
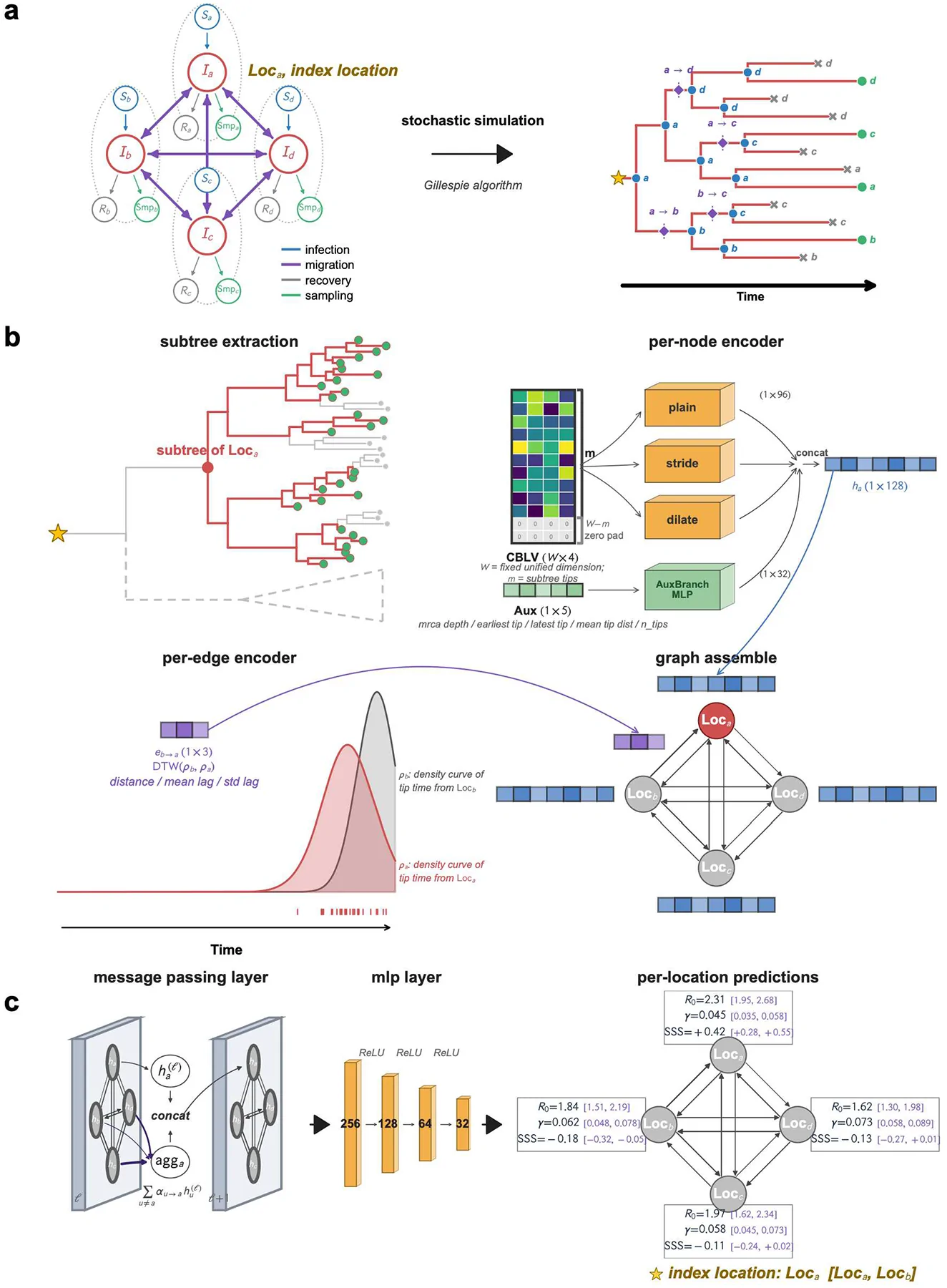
Overview of the STEPHY framework for inferring region-specific epidemic parameters from phylogenetic trees, illustrated using a 4-region system. **a. Data generation:** A stochastic SIR model with inter-region migration is simulated under the Gillespie algorithm to generate a sampled multi-region outbreak phylogeny. Each region includes susceptible (S), infected (I), recovered (R), and sampled (Smp) compartments, with migration connecting all pairs of regions. The example phylogeny is color-coded by event type: infection events are shown in blue, removals in grey, sampling events in green, and migration events as purple diamonds. The index case is seeded in $Loc_{a}$ and marked with a gold star. **b. STEPHY encoder pipeline:** Both the per-node and per-edge encoders begin with subtree extraction. For a chosen region, the corresponding induced subtree is extracted from the full phylogeny. Using ${Loc}_{a}$ as an example, the per-node feature $h_{a}$ is computed from its induced subtree through two parallel heads: a CBLV representation processed by three convolutional branches, plain, strided, and dilated, and an auxiliary head that passes five tree statistics, TMRCA, earliest tip time, latest tip time, average branch length, and number of tips, through a multilayer perceptron. The outputs of the two heads are then concatenated. Similarly, the directed edge feature $e_{b\to a}$ is computed by extracting the induced subtrees for $Loc_{a}$ and $Loc_{b}$, estimating their tip-time density curves, $\rho_{a}$ and $\rho_{b}$, applying dynamic time warping (DTW) between the two curves, and summarising the alignment using the DTW distance, mean lag, and lag standard deviation. Applying the node procedure to all 4 regions and the edge procedure to all 12 ordered region pairs populates the directed $K_{4}$ graph. **c. Inference:** An edge-aware attention message-passing layer updates each node embedding by aggregating messages from its three neighbors, weighted by learned attention coefficients α, and concatenating the result with the node’s previous-layer embedding. The updated embedding is passed through a multilayer perceptron head, producing per-region predictions of the effective reproduction number $R_{0}$, recovery rate γ, and source-sink score SSS, each accompanied by its 95% conformal prediction interval. The model also predicts the index location and its 95% conformal prediction set.

**Figure 2.**
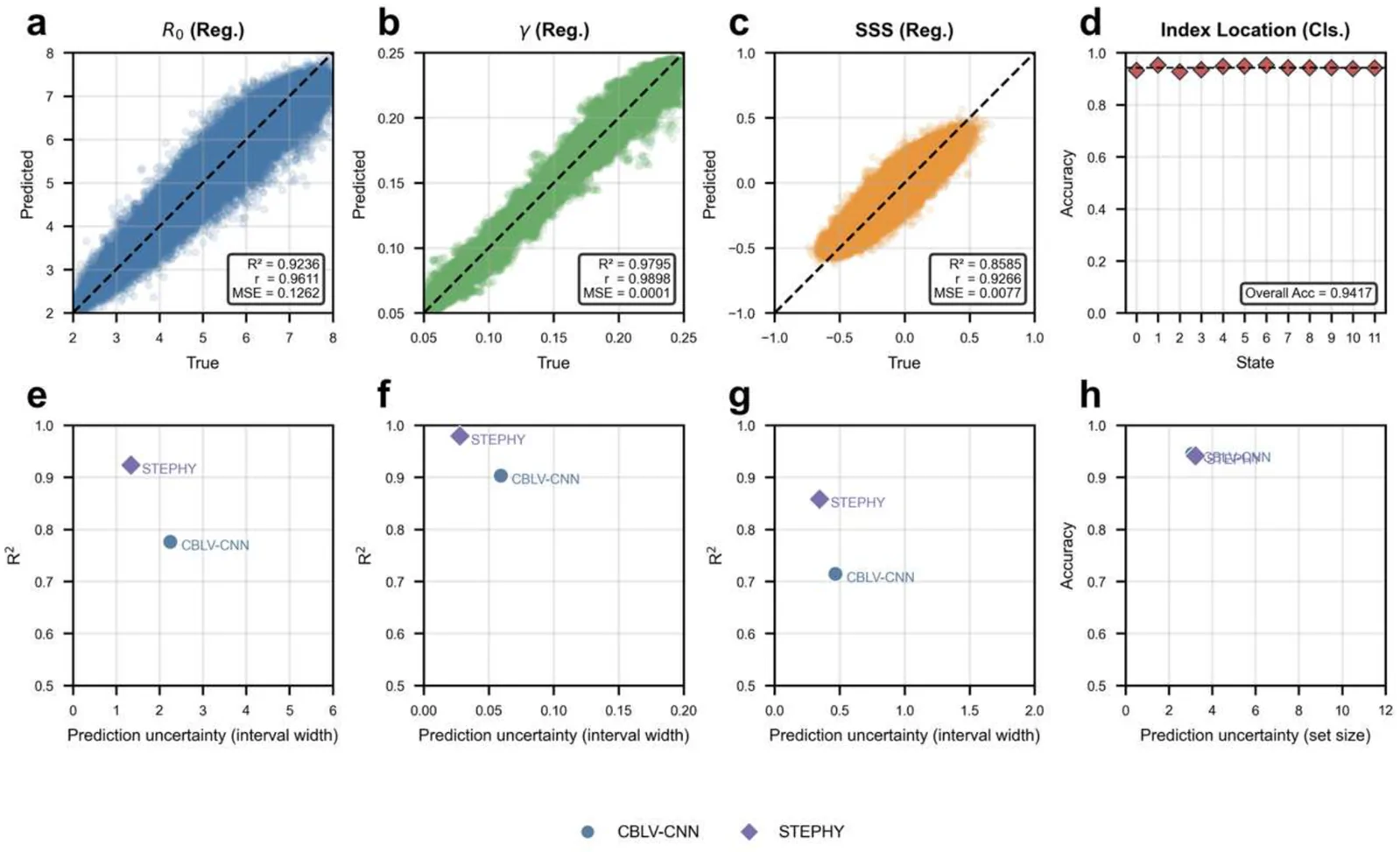
Simulation-based evaluation of STEPHY across regression and classification targets **a-d**. Per-target performance of STEPHY on the held-out test set. **a-c**. Predicted versus true values for the three regression targets: $R_{0},\gamma$, and SSS. The dashed line marks the identity line. Insets report the coefficient of determination $\left(R^{2}\right)$, Pearson correlation (r), and mean squared error (MSE), all computed using the median quantile prediction from the CQR head. **d**. Per-region accuracy for the index-location inference. The dashed horizontal line marks the overall accuracy reported in the inset. **e–h**. Joint comparison of median-prediction accuracy and conformal-prediction uncertainty for STEPHY and CBLV-CNN. For regression targets (**e–g**), the y-axis shows $R^{2}$ of the median quantile prediction, and the x-axis shows the mean conformal interval width computed on the calibration split using CQR. For classification (**h**), the y-axis shows the accuracy, and the x-axis shows the mean prediction-set size computed on the calibration split using RAPS. Points in the upper-left corner indicate models that combine accurate median predictions with tighter predictive intervals or smaller prediction sets.

**Figure 3.**
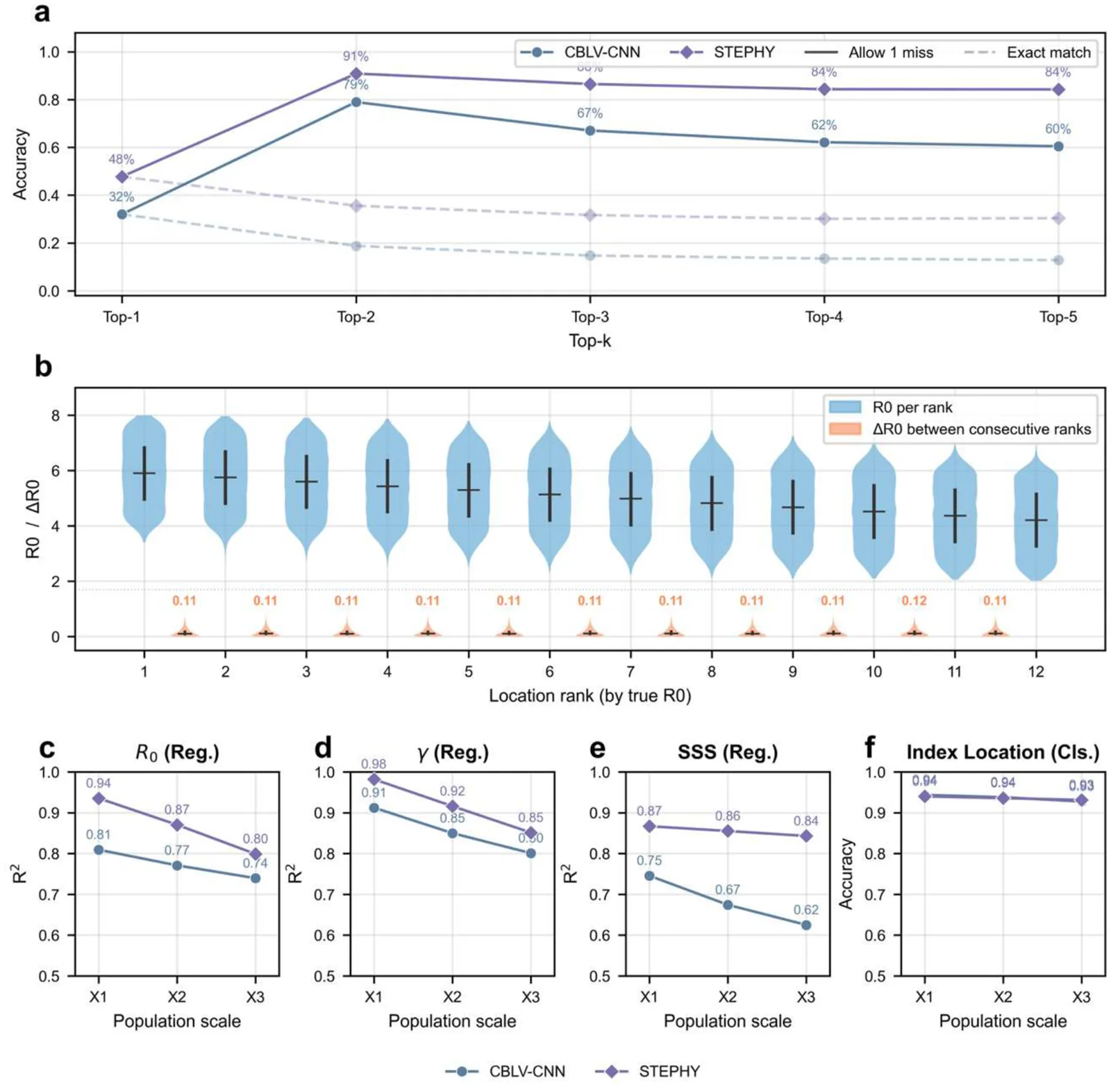
Stress-testing STEPHY on $R_{0}$ ranking and population-scale generalization. **a.** Top-k accuracy for identifying regions with the highest $R_{0}$ across CBLV-CNN and STEPHY. Solid lines allow one mismatch; dashed lines indicate exact-match accuracy. **b**. Distribution of true $R_{0}$ by descending rank across all test outbreaks. Blue violins show $R_{0}$ values at each rank, and orange violins show the gaps between adjacent ranks. Thick vertical lines indicate the interquartile range, and horizontal lines indicate the median. **c–f**. Generalization performance under population rescaling relative to the training range (5K–50K). Models trained on the default population range were evaluated on test sets with population scaled by 1, 2 and 3 and sampling rate reciprocally scaled by 1, 0.5 and 0.33, respectively. $R^{2}$ is shown for $R_{0}$ (**c**), γ (**d**) and SSS (**e**); classification accuracy is shown for index location (**f**).

**Figure 4.**
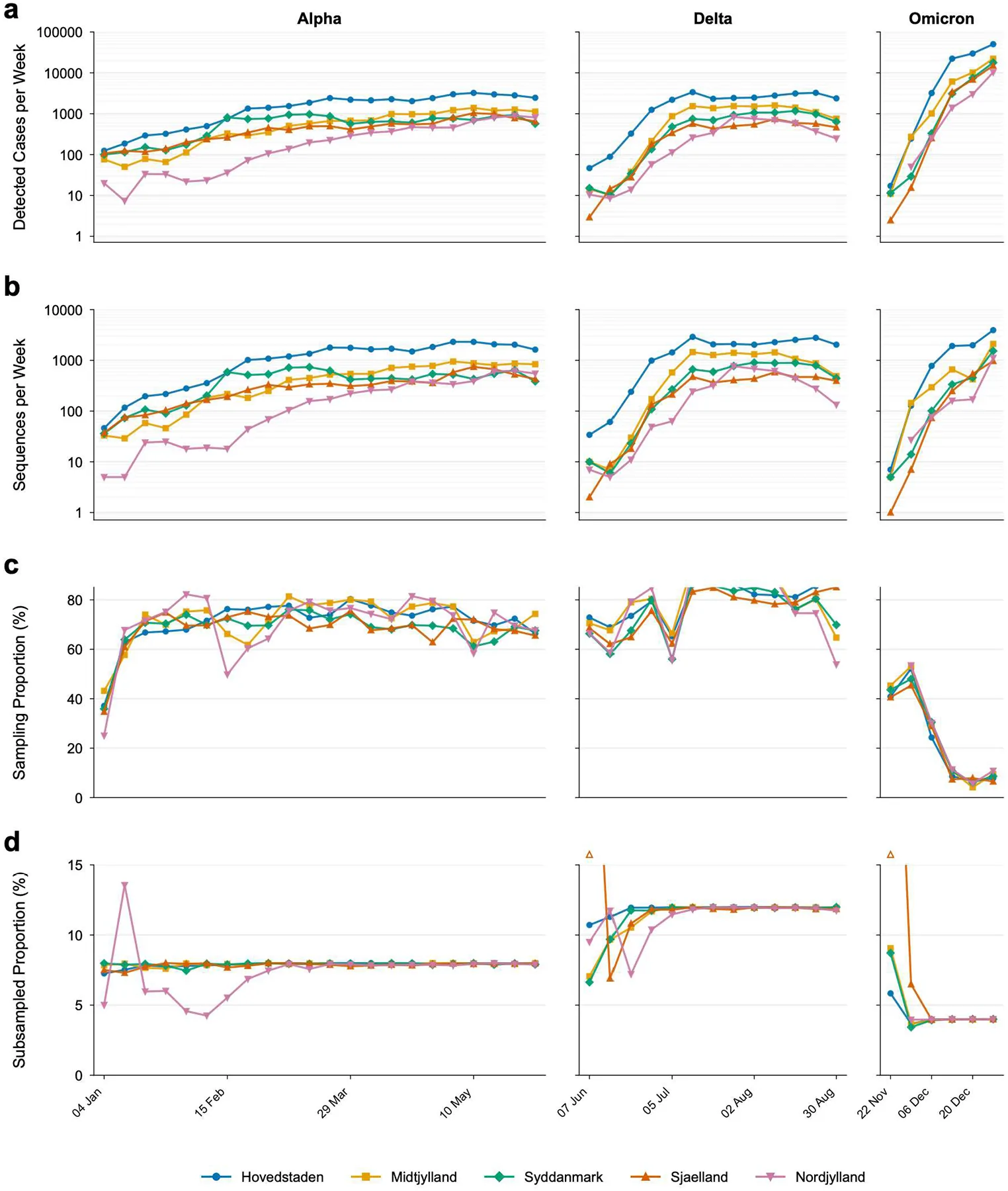
Sequencing coverage and proportional subsampling of SARS-CoV-2 variants in Denmark, 2021. **a**. Estimated weekly detected cases by variant and region, calculated as the product of SSI-confirmed regional case counts and GISAID-derived variant proportions. **b**. Raw weekly sequence counts from GISAID, filtered to records with complete collection dates and assigned to one of five Danish regions. **c.** Sequencing sampling proportion before subsampling, defined as the ratio of available sequences (**b**) to estimated detected cases (**a**), expressed as a percentage. **d.** Sequencing sampling proportion after proportional downsampling. Each (variant, epiweek, region) cell was capped at a variant-specific baseline: 8% for Alpha (W01–W22), 12% for Delta (W23–W35) and 4% for Omicron (W47–W52).

**Figure 5.**
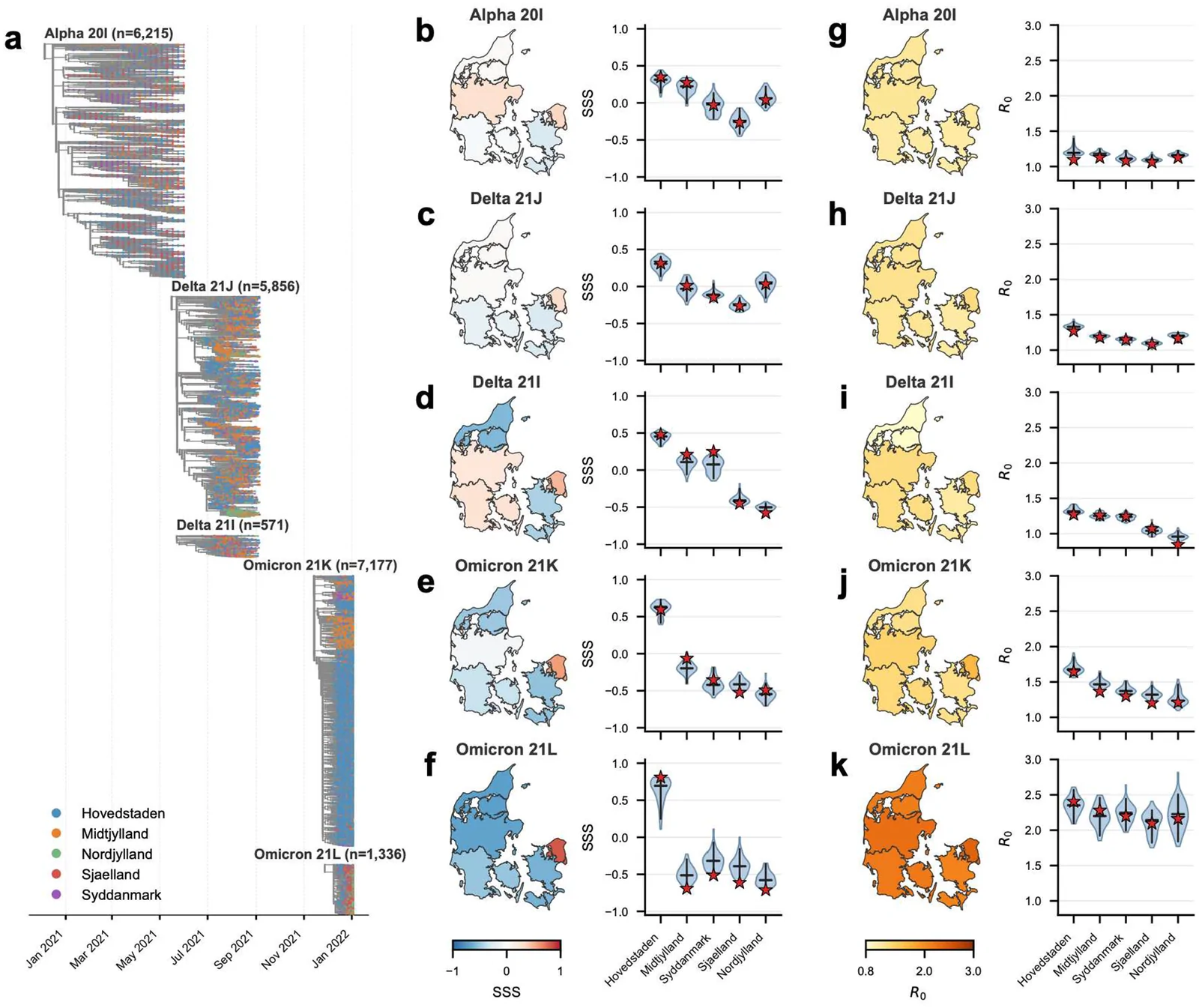
Phylogeographic analysis of SARS-CoV-2 variant spread across Danish regions. **a.** Time-scaled phylogenies of five SARS-CoV-2 lineages circulating in Denmark, ordered chronologically from top to bottom: Alpha 20I, Delta 21J, Delta 21I, Omicron 21K, and Omicron 21L. Tips are colored by region. Vertical joining branches are scaled by descendant tip count. **b–f**. Per-lineage STEPHY inference of source–sink score (SSS) across the five Danish regions. Each panel pairs a choropleth map on the left with a violin plot on the right. Choropleths show SSS predicted from the maximum-likelihood time tree, with one value per region. SSS values near +1 indicate net sources, whereas values near −1 indicate net sinks. All choropleths use a shared color scale. To assess sensitivity to phylogenetic uncertainty, STEPHY was re-applied to 40 bootstrap time trees. Blue violins show the resulting per-region distributions, black bars indicate medians, and red stars indicate the maximum-likelihood tree estimates shown in the choropleths. Tight violins centered on the red stars indicate estimates that are stable under topological uncertainty. **g–k**. As in b–f, but for regional basic reproduction number, $R_{0}$.

**Table 1.** Per-tree forward-pass latency on the held-out test set (n = 5,999 trees, 12 sampled regions each). Measurements were performed with batch size 1 in fp32 on a single Intel Xeon Gold 6242 CPU core at 2.80 GHz using PyTorch 2.4 and DGL 2.4 in single-threaded mode. Pearson r is the correlation between per-tree latency and tree size (range 580–5,000).

Task	Params	Mean (ms)	Min (ms)	Median (ms)	Max (ms)	Pearson r
$R_{0}$ (Reg.)	60,192	5.85	5.70	5.77	8.28	−0.009
y (Reg.)	60,192	5.86	5.70	5.79	8.91	−0.001
SSS (Reg.)	60,192	5.85	5.69	5.77	9.98	+0.033
Index Location (Cls.)	60,126	5.95	5.75	5.90	10.15	+0.020

**Table 2.** Per-tree forward-pass latency for Denmark SARS-CoV-2 maximum-likelihood trees. Latency was measured for five trees representing lineages 20I, 21J, 21I, 21K, and 21L, with 6,215, 5,856, 571, 7,177, and 1,336 tips, respectively. Each tree included five sampled regions. Measurements were performed with batch size 1 in fp32 on a single Intel Xeon Gold 6242 CPU core at 2.80 GHz using PyTorch 2.4 and DGL 2.4 in single-threaded mode.

Task	Params	Alpha 20I (ms)	Delta 21J (ms)	Delta 21I (ms)	Omicron 21K (ms)	Omicron 21L (ms)
$R_{0}$ (Reg.)	60,126	18.99	18.92	18.93	18.94	18.96
γ (Reg.)	60,126	18.99	19.00	19.02	18.98	18.98
SSS (Reg.)	60,126	19.03	19.00	19.00	18.96	18.96
Index Location (Cls.)	60,126	18.95	18.95	19.00	18.98	18.98

## Data Availability

The phylogenetic intermediates used in the Denmark analysis are deposited at https://doi.org/10.5281/zenodo.21766003. Genome sequences were obtained from GISAID. Accession identifiers and the corresponding laboratory acknowledgement table are provided in the case-study repository.

## Code availability

A documented implementation of STEPHY is available at https://github.com/leke-lyu/STEPHY. The repository includes workflows for outbreak simulation, model training, uncertainty quantification, and downstream inference, allowing adaptation to different pathogens, population structures, and surveillance settings. Code and analysis files for the empirical application to SARS-CoV-2 transmission in Denmark are available at https://github.com/leke-lyu/stephy-denmark. Trained model weights are deposited at https://doi.org/10.5281/zenodo.21766065.
